# The greater the pleiotropic effects, the greater the benefits - cannabis as a “biopsychosocial” drug: a mixed-methods study on chronic non-cancer pain

**DOI:** 10.1186/s42238-026-00440-w

**Published:** 2026-04-21

**Authors:** Inês C.A. Pombeiro Stein, Franziska Fitzner, Martin Dusch, Christian S. Kessler, Jan Moritz Fischer, Farid-Ihab Kandil, Felix Freigang, Matthias Karst

**Affiliations:** 1 Department of Anesthesiology and Intensive Care Medicine, Pain Clinic, Hannover Medical School, Hannover, Germany; 2 PRACTIS Clinician Scientist Program, Dean’s Office for Academic Career Development, Hannover Medical School, Hannover, Germany; 3 Pain Centre, Department of Anesthesiology, Intensive Care Medicine, and Pain Medicine, Medical Faculty Mannheim, University Medical Centre Mannheim, University Heidelberg, Mannheim, Germany; 4 Department of Integrative Health Care and Prevention, University of Augsburg & University Hospital Augsburg, Augsburg, Germany; 5 Department of Internal Medicine and Nature-based Therapies, Immanuel Hospital Berlin, Berlin, Germany; 6 Institute of Social Medicine, Epidemiology and Health Economics, Charité-Universitätsmedizin Berlin, Berlin, Germany; 7 Corporate Member of Freie Universität Berlin and Humboldt-Universität zu Berlin, Berlin, Germany; 8 University Hospital Bonn, Institute for Medical Biometry, Informatics, and Epidemiology (IMBIE), University of Bonn, Bonn, Germany; 9 Center for Health Services Research, Faculty of Health Sciences Brandenburg, Brandenburg Medical School Theodor Fontane, Rüdersdorf, Germany

**Keywords:** Cannabis, Cannabinoids, Chronic pain, Patient reported outcomes, Mixed methods, Qualitative research

## Abstract

**Background:**

Against the background of widely inconsistent data from randomized controlled trials (RCT), the use of cannabis-based medicines (CBM) from the perspective of patients with chronic non-cancer pain (CNCP) was described.

**Methods:**

Based on a purposive/convenient sampling, patients were recruited from the Pain Clinic of Hannover Medical School who had been using CBM prescribed by a doctor for at least 6 months. The patients discussed their experiences with CBM in semi-structured individual interviews. The interview transcripts were coded and analyzed using a modified grounded theory approach with the help of MAXQDA^®^. In addition, the Treatment Satisfaction Questionnaire with Medication (TSQM) was used.

**Results:**

Theoretical saturation was reached after 32 interviews. Open and selective coding revealed the overarching phenomenon of “subjective pain experience under CBM therapy”, with one of the main themes being the “effect of CBM”. This revealed the categories “effect on pain” and “psychological” and “somatic effect”. The most important concepts were “pain intensity”, “pain management”, “stress management”, “musculoskeletal system”, and “sleep quality.” Constructing a theoretical framework 4 groups of responses to CBM treatment were identified. The focus is either on (I) *pain reduction*, (II) *pain coping*, (III) *reduced stress* or (IV) *multidimensional aspects*. When this classification was applied to topic of quality of life (QOL), the greatest effectiveness and highest overall satisfaction were found in group (IV). Mixed methods showed a continuous increase in the perceived effectiveness of CBM on pain-centered complaints from group (I) to (IV).

**Conclusions:**

In line with the biopsychosocial understanding of chronic pain, it appears that those CNCP patients who benefit most from CBM are those who show the most far-reaching effects on both a physical and psychological level. The pleiotropic effects of CBM may be responsible for this. Based on these results, interdisciplinary prospective research appears sensible and necessary to further and systematically investigate this clinically relevant topic.

**Trial registration:**

This study was registered with the German Clinical Trials Register (DRKS00037434), after approval by the local ethics committee (No. 8391_B0-K_2019).

**Supplementary Information:**

The online version contains supplementary material available at 10.1186/s42238-026-00440-w.

## Background

Chronic pain, i.e. pain that lasts longer than three months, occurs frequently and can have a negative impact on individual quality of life (QOL) and society (Treede et al. 2019). As an epidemiological analysis in Germany has shown, it is possible to speak of an independent pain disorder if chronic pain is associated with considerable suffering and functional impairment (Häuser et al. 2014). This interpretation was defined by the current ICD-11 classification as chronic primary pain (Barke et al. 2022). There are ongoing or biographical risk factors that favor the occurrence and continuation of chronic pain that have been identified through population-based studies: female gender, older age, socio-economic status, cultural background, lifestyle, employment status and occupational factors (including injury) (Hecke et al. 2013) as well as history of childhood adversity and psychological trauma (Nicolson et al. 2023, Kodila et al. 2024, Nelson et al. 2020, Agorastos et al. 2019). As shown in a cross-sectional study of 914 patients with chronic pain from a German university hospital outpatient clinic, there even appears to be a dose-response effect, which shows that the greater the psychological trauma, the more intense the symptom burden (Manuel et al. 2023). Furthermore, the observed increase in pain area and widespread pain, as well as the impact on clinical endpoints such as pain intensity, sleep disturbance, symptom burden, disability and stress in the most severely affected group, are consistent with the concept of central sensitization in patients with post-traumatic stress disorder (PTSD) (Manuel et al. 2023). Central pain sensitization refers to increased neuronal signaling in the central nervous system (CNS) that leads to hypersensitivity to pain, which manifests clinically as diffuse, widespread pain that is out of proportion to what would be expected based on the existing presumed source of nociception (Nijs et al. 2021). Along with a number of biopsychosocial aspects that correspond to the multidimensional nature of chronic pain, the new pain classification “nociplastic pain” was established by the International Association for the Study of Pain (IASP) (Nijs et al. 2021).

Chronic stress, which is therefore a risk factor for the development of chronic pain and is associated with the state of central pain sensitization, increases the perception of pain (Schaffer et al. 2023). A dysfunctional connectome in the corticomesolimbic system is considered to be the pathophysiological correlate of such centralized pain (Vachon-Presseau et al. 2016, Motzkin et al. 2026). These brain regions involved in pain and stress processing are densely populated with cannabinoid receptors (Breivogel and Sim-Selley 2009, Hu and Mackie 2015, Morena et al. 2016). Thus, the endocannabinoid system (ECS) appears to play a crucial role in cognitive and affective pain processing. This is supported by clinical and experimental data showing that CBM alters the affective component of pain rather than pain intensity (Vita et al. 2018, Aviram et al. 2021). Furthermore, it has been shown that patients with lumbar sciatica experienced greater pain relief from THC the more pronounced the dysfunction of the corticomesolimbic system was (Weizman et al. 2018). In patients with fibromyalgia syndrome, the administration of a THC-dominant cannabis extract led to an enhancement of central pain inhibition (offset analgesia) and thus to significant pain relief (Agbaria et al. 2025).

As already indicated, CBM may have effects other than simply reducing pain. This is indicated by observational studies on the one hand and qualitative studies on the other. A systematic review and meta-analysis (SRMA) by Bialas et al. (Bialas et al. 2022), which examined long-term observational studies on CBM (mainly by inhalation) and included data from around 2,500 patients, showed highly significant improvements not only in terms of pain intensity, but also in terms of function, sleep quality, depression, anxiety and general QOL. In addition, around 16% of patients were able to stop their opioid medication during CBM therapy (Bialas et al. 2022). A recent SRMA showed no differences in the efficacy of CBM in terms of pain reduction and improvement in function and sleep between nociceptive, neuropathic, cancer and non-cancer pain (Wang et al. 2021). In a large observational study initiated by the Federal Ministry for Drugs and Medical Devices as part of the prescription option for CBM in Germany since 2017, it was found that around 8000 out of 14,180 patients were prescribed CBM only for the three ICD-10 diagnoses chronic uncontrollable pain (R52.1), other chronic pain (R52.2), and chronic pain with somatic and psychological factors (F45.41) (Bundesinstitut 2022. The diagnoses are rather imprecisely defined, which could indicate that CBM addresses not only pain but also other overarching factors. A recently published qualitative study in which patients with chronic pain were interviewed showed that patients under CBM perceived highly complex changes, which the authors referred to as “restored self”, although in some cases significant pain persisted (Lavie-Ajayi and Shvartzman 2019). This example illustrates that the complex effects of CBM on perception, psychomotor functions, motivation, mood, relaxation, and sleep lead to a positive treatment outcome only when they interact with one another. In pharmacology, the diverse effects triggered by a substance are referred to as pleiotropic effects – a concept believed to apply to CBM as well (Karst 2024, Candeloro et al. 2025).

In the course of the years, around 60 randomized clinical trials (RCTs) on the efficacy and safety of CBM for chronic pain have been published. In order to evaluate the results more accurately, approximately the same number of systematic reviews and meta-analyses (SRMAs) have been published since 2010 (Eisenberg et al. 2022). This unfavorable ratio, combined with very different statements ranging from clear evidence of efficacy to the complete opposite, has mainly remained unchanged to this day (Chou et al. 2020). The RCTs included in the SRMAs are extremely heterogeneous in every respect, particularly about the specific CBM used, the galenics, the pain situation and the outcome parameters investigated (Eisenberg et al. 2022). This is also reflected in the reported values for the “number needed to treat for benefit“ (NNTB), which show a wide range and vary between 2 and 24 (Campbell et al. 2019). Against this backdrop, many SRMAs conclude that the evidence is weak or insufficient and that such meta-analyses therefore do not provide sufficient information about which interventions are most appropriate for patient care (Ioannidis 2016). Furthermore, there often remains a strong impression of subjectivity permeating supposedly objective quantitative methods (Ioannidis 2016, Greiner 1962).

In order to better understand these phenomena surrounding the use of CBM for chronic pain, semi-structural in-depth interviews were conducted in the pain outpatient clinic of Hannover Medical School with chronic non-cancer pain (CNCP) patients who had been taking CBM for at least six months. The aim was to use the results to generate new hypotheses about the clinical effects of CBM in patients with CNCP. These hypotheses could also influence the selection of outcome parameters for RCTs in chronic pain.

## Methods

The inclusion criteria for participants were an age of ≥ 18 years, voluntary participation, and the use of CBM for a period of at least 6 months due to CNCP. In Germany, CBM is reimbursed by health insurance providers only if patients are severely impaired by their pain condition and other therapeutic approaches have failed. This means that, in addition to certain specific symptoms and syndromes (Table 2), all patients suffered from long-lasting chronic pain associated with the ICD-10 diagnoses R52.2 or F45.41, which involve nociplastic components of pain. Further information about our cohort is provided in Table 1, and Table S1 in the Supplement. As a rule, the CBM dose is titrated over several weeks (Bashkar et al. 2021). Sometimes it is necessary to switch to a different type of CBM. To ensure that patients had reached a stable condition and were able to provide a meaningful assessment of their treatment, we therefore decided to determine their eligibility for the study only after they had been taking CBM for at least six months.

**Table 1:** Highest level of education: Low = Junior High School Diploma; Medium = Middle School Diploma, Vocational Training, Secondary School Diploma; High = High School Diploma, College, Academic Degree. Baseline sociodemographic characteristics of the participating patients

Sex, *n* (%)	
Women Men	15 (46.9) 17 (53.1)
Age, years	
Mean Median Youngest Oldest	52.4 51 22 77
Marital status, n (%)	
Single Married Separated/Divorced Widowed	12 (37.5) 10 (31.3) 7 (21.9) 3 (9.4)
Persons in household, n (%)	
Living alone With 1 other person With 2 other persons With 3 other persons	16 (50) 10 (31.3) 2 (6.3) 4 (12.5)
Children, n (%)	
No 1 child 2 children 3 children	10 (31.3) 11 (34.4) 8 (25) 3 (9.4)
Education level, n (%)	
Low Medium High	6 (18.8) 18 (56.3) 8 (25)
Employment status, n (%)	
Retired/Disability pension Employed Unemployed	21 (65.6) 9 (28.1) 2 (6.3)
Duration of CBM use, months	
Mean Median Shortest Longest	41 20 6 52
Other medications from pain clinic, n (%)	
Yes No	18 (56.3) 14 (43.8)

The design of our exploration was a prospective cross-sectional study with a mixed methods approach, consisting of a qualitative part (partially structured patient interviews) and a quantitative questionnaire on medication satisfaction (German TSQM, version 1.4).

We decided on a qualitative approach since, as previously discussed, there is a gap between patient reported outcomes and systematic evidence, and we considered that a quantitative approach may not be suited (Foley and Timonen 2015) to best assess the influence of CBM on sleep quality, mood and life quality, which arguably contribute significantly to a positive overall experience for patients. We used grounded theory as an analytical method for the qualitative findings, which is an inductive process and generates hypotheses from the data, as opposed to deductive techniques, which are traditional in quantitative research and analyze data to refute or confirm hypotheses (Chapman et al. 2015). The grounded theory method was developed by Barney Glaser and Anselm Strauss in 1967, and there are three different approaches that are commonly used (Singh and Estefan 2018). The study team chose Strauss and Corbin’s perspective because it provides a clear and systematic analysis (Singh and Estefan 2018). The technique of Corbin and Strauss contains three steps: open coding, axial coding and selective coding (Singh and Estefan 2018). During open coding the interviews are read, and any meaningful text passage is labelled with a code. A code is a keyword or a catchphrase that summarizes the content without interpretation. During the analytic process text passages from different interviews are compared to each other and are given the same code, if applicable (Singh and Estefan 2018). In our study we used the four-step coding tool to sort codes into (sub-)concepts, categories and themes to present the data in a structured way (Qureshi and Ünlü 2020). In the axial coding data are reassembled to identify relationships between categories and concepts forming a framework called paradigm model. In the selective coding the insights gained from the paradigm model are incorporated into a coherent descriptive presentation about the overarching phenomenon. The goal is to develop a comprehensive theory, that still acknowledges differences in the experience (Singh and Estefan 2018).

Patients from the Pain Clinic who got prescribed CBM for at least 6 months were recruited following the principles of purposive and convenience sampling and data were collected until saturation was achieved (Moser et al. 2018). To ensure the best possible quality of evidence conducting and reporting the qualitative interviews, we used the 32-item Consolidated Criteria for Reporting Qualitative Research (COREQ)-checklist (Tong et al. 2007).

For the development of an interview guideline, the study team analyzed existent literature and decided on partially structured interviews which were designed based on the framework proposed by Kallio and colleagues (Kallio et al. 2016). Pharmacological, psychological and social factors were deemed important and the study team determined which topics should be covered in the interview: the circumstances of therapy beginning, related expectations, occurred changes since therapy beginning, the effect of CBM and its effect on everyday life as well as QOL. When defining the order of questions, the study team followed the recommendations of Moser and colleagues and decided on primarily asking “what” and “why” questions, followed by “how” questions for deepening the conversation (Moser et al. 2018).

The researchers determined a logical order of questions, starting with the beginning of therapy and moving on to the effects of CBM during the course of the interview. The question about the effect of CBM on pain was posed towards the end, as to not solely focus the questioning on this topic. It was decided that the order of the questions could be altered in the interviews, to allow an open dialog instead of a strict question-answer-catalogue (Moser et al. 2018). The team then formulated follow-up questions and key points and added them to the interview guideline. It was also possible for the interviewer to adapt the questions according to the individual situation through probes and prompts as well as verbal and non-verbal signs (Moser et al. 2018). After internal testing, the final interview guide was used with participants 1–25 and following an interim evaluation by the study team, some changes were made to the guide addressing additional questions about experiences of relaxation methods and psychotherapy. The updated version was used with participants 26–32. Both versions of the interview guideline can be found in the supplemental data.

This study was registered with the German Clinical Trials Register (DRKS00037434). After approval by the local ethics committee (No. 8391_B0-K_2019) interviews took place between September 2020 to March 2021. Prior to the interviews, written consent was obtained from the patients; patients were free to withdraw from the study at any time or refuse to answer questions if they were uncomfortable. Twenty-three interviews were conducted at the pain clinic at Hannover Medical School, one interview took place at the work venue from one participant, and eight patients were interviewed by telephone.

All interviews were recorded on tape, without systematically documenting nonverbal behavior (e.g., through video recordings), transcribed verbatim, and anonymized; the transcripts were not corrected or commented by the patients. After transcription, the transcripts were reviewed sentence by sentence using MAXDA^®^ and analyzed for the literal meaning of the text (Hussy et al. 2013). Care was taken to ensure that the code accurately reflects the content of the statement and does not involve any interpretation. In vitro and in vivo coding was then performed (Harris 2015). In vivo coding uses purely descriptive and content-oriented terminology, while in vitro coding uses more abstract terms, but still without interpretation (Harris 2015). Examples of codes from our study are:

In vivo coding: “Well, I can definitely fall asleep better.” (UE118) ◊ Falling asleep.

**Fig. 1:**
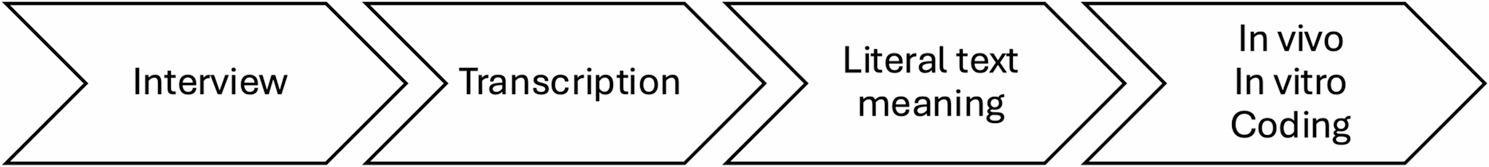
Workflow for the generation of codes during the qualitative research process (Hussy et al. 2013, Harris 2015)

In vitro coding: “Well, but… ultimately… mentally it also had many positive effects, which in turn means that, as I said, I want to have goals again relatively quickly. So I said: ‘I want to work again, I want to tackle this at some point. I’m not so old that I’m going to give up now and say: Here, I’ve lost.’ So that had a big impact on me.” (II017)◊ Self-efficacy (Fig. 1).

**Fig. 2:**
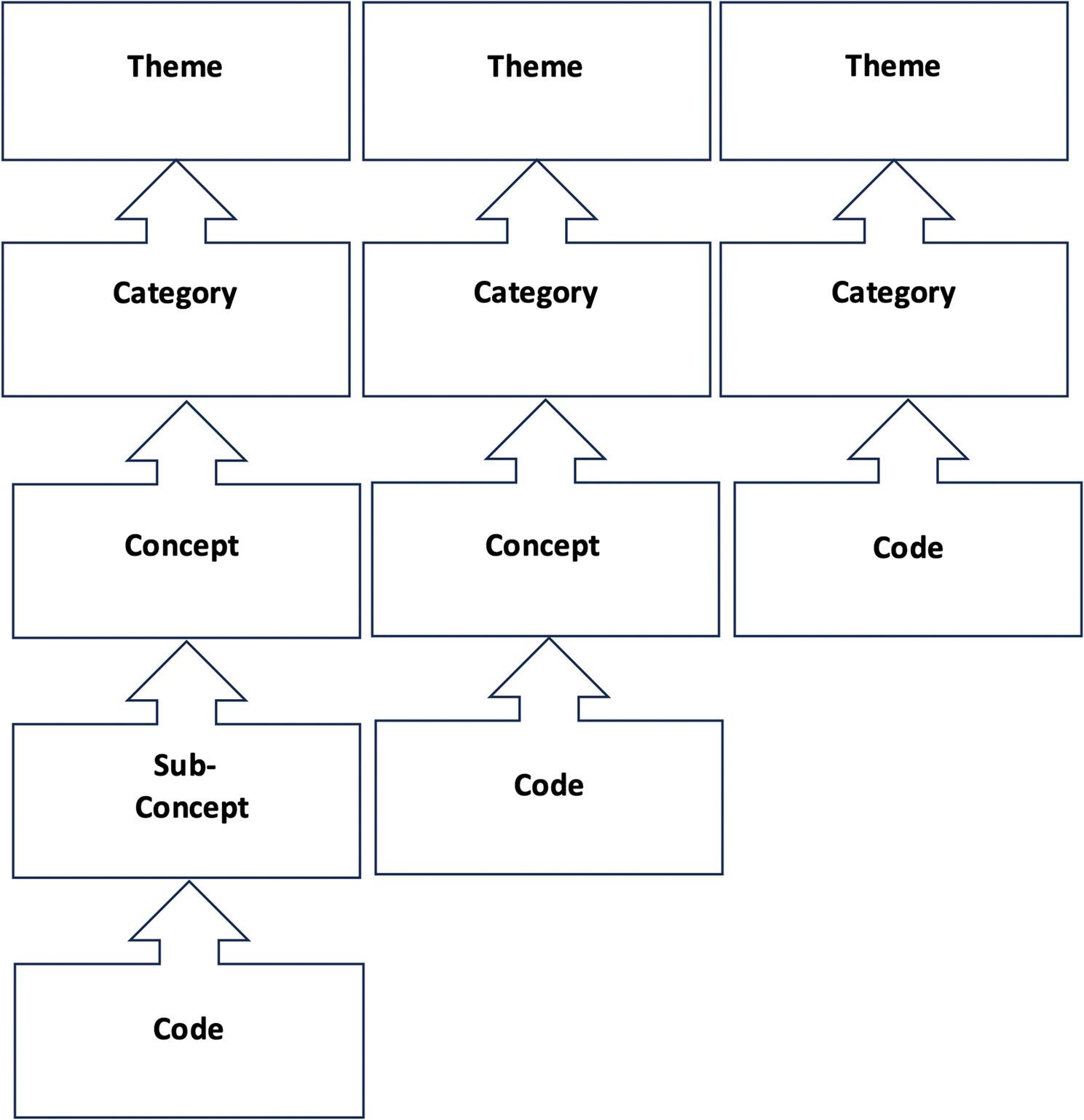
Key terms inside the coding instrument. Adaptation and modification from Qureshi and Ünlü 2020

The four step coding tool proposed by Qureshi and Ünlü (Qureshi and Ünlü 2020) was adapted to the present study and used to analyze the data. As some topics were discussed more intensively than others in our study, in some cases there were more or less than four levels. If our data induced five steps, a subordinate concept was introduced. If only three steps were evident, concepts were excluded and categories directly extracted (Fig. 2).

Initially, open coding was employed to classify statements from different patients which were similar in terms of content and label them with the same code. Thematically related codes were then summarized into (subordinate) concepts. Concepts were in turn grouped into categories and categories into themes. Axial coding was then applied by using the paradigm model from Corbin and Strauss (Singh and Estefan 2018). This model helps to relate data to one another by defining the overarching phenomenon as “pain perception during CBM therapy” and dividing the themes into underlying conditions (in our study: prescripton, initial state), context (in our study: healthcare system, factors that have influenced patients’ attitudes toward CBM, coping strategies), intervening conditions (in our study: user experiences of CBM therapy), effect (in our study: effects of CBM therapy on different domains), and consequences (in our study: impact of CBM on QOL) (Table S2 in the supplement). At last, selective coding was applied to a comprehensive theory that characterizes the subjective pain experience during CBM-therapy, while still considering the differences in experiences. Codes were only applied once for each patient.

MAXQDA^®^ (2022) software was used for data management (data organization and visualization). After careful analysis of the themes, the codes attributed to each patient were considered and four groups were formed. Since each code was used only once for each patient, the only relevant factor was whether the code was used, not how often. It is also important to note that some patients did not experience any specific effects, and therefore certain codes are missing (see the Results section).

All participants were interviewed by the same researcher (FraF), who also transcribed and analyzed the interviews. In a further step, three selected interviews were analyzed independently by three researchers (MK, JMF, F-IK) who were not involved in the interviews. Results were examined and discussed by the entire research team to achieve higher intercoder reliability.

Additionally, to the interview, each patient received a questionnaire about satisfaction with medication (German Treatment Satisfaction Questionnaire for Medication, TSQM, version 1.4), comprising questions about effectiveness, side effects, convenience and global satisfaction ( 2015, Atkinson et al. 2005, Atkinson et al. 2004). Each section was individually assessed and given a value between 0 and 100 (100 representing total satisfaction). The questionnaire included 14 questions. The areas effectiveness, convenience and overall satisfaction include three questions each. Regarding side effects, the patients were first asked if they suffered any side effects; if the answer was no, it was attributed a value of 100; if the answer was yes, there were four follow-up questions posed. Analysis has been done according to the formula that is described elsewhere (Atkinson et al. 2005, Atkinson et al. 2004, Bharmal et al. 2009).

Finally, the mixed methods approach was achieved by combining qualitative and quantitative data. Regarding effectiveness, side effects, convenience, and overall satisfaction, which were determined using the German TSQM, differences were sought between the 4 groups that had been formed after analysis of the qualitative data.

## Results

In this study, 32 patients were included from the Pain Clinic of the Department of Anesthesiology and Intensive Care, Hannover Medical School, who were between 22 and 77 years old (mean age 52.4 years) and interviewed after they had signed the consent form.

**Fig. 3:**
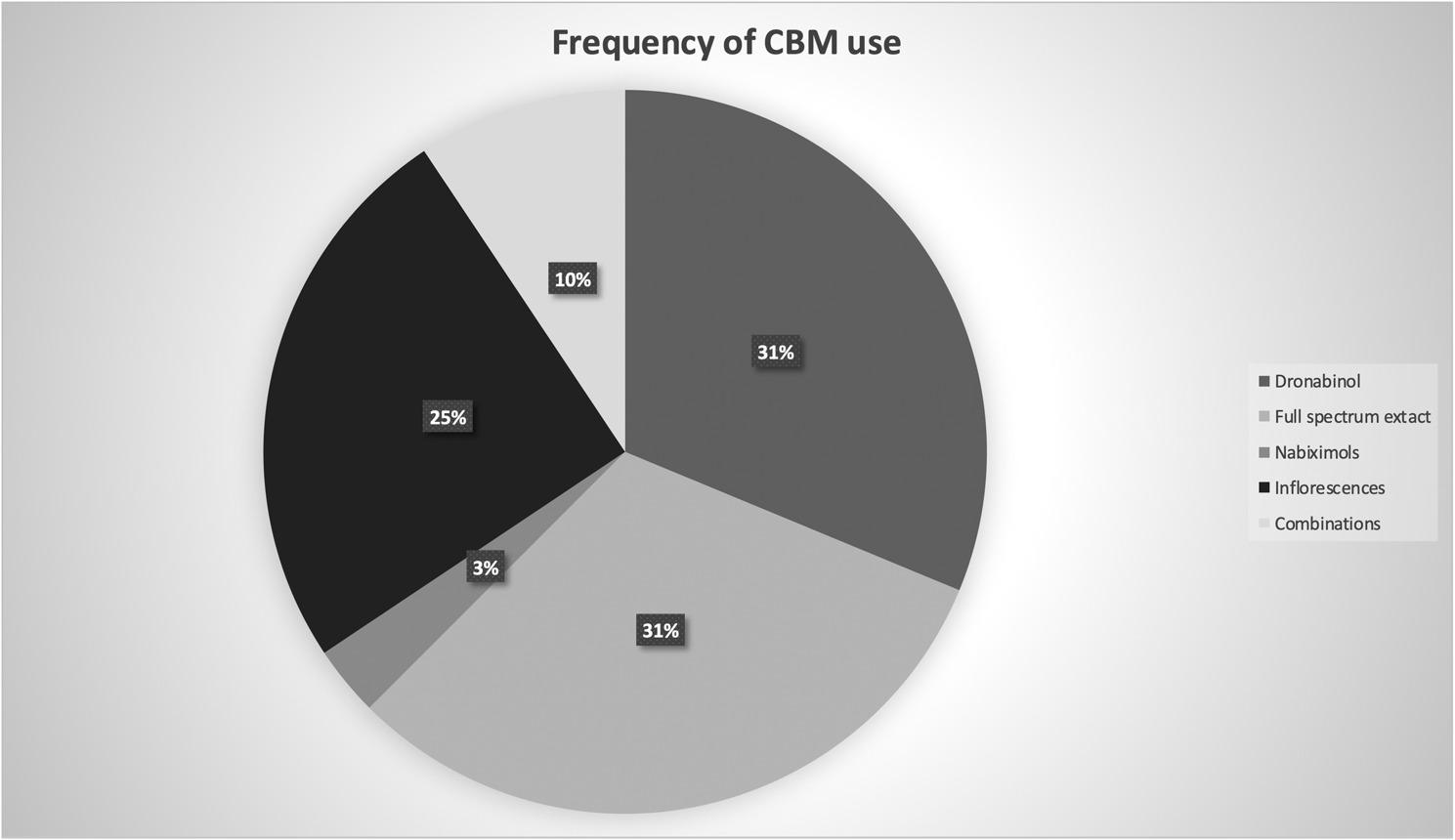
Frequency of cannabis-based medication (CBM) use

The shortest interview lasted 16 min, the longest 88 min; the average interview length was 35 min. Patients received CBM between 6 and 52 months mainly for neuropathic pain, muscle/joint pain, cramps/spasticity, or headaches (Table 2), in 56.3% in addition to other pain medication. Dronabinol (31%), full spectrum extract (31%), and inflorescences (25%) were most often prescribed (Fig. 3). In Table 1, a summary of the demographic characteristics of the interviewed patients is depicted.

**Table 2:** Reasons for prescribing CBM. From the 32 interviews, 13 different symptoms were mentioned a total of sixty times. This shows that some patients cited several symptoms and syndromes as reasons for prescribing CBM

Symptoms/Syndromes	Frequency of codes
Neuropathic pain	17
Muscle/joint pain	10
Cramps/spasticity	8
Headache (tension-type)	4
Migraine	4
Psychological symptoms	3
Polyneuropathy	3
Posttraumatic Stress Disorder	3
Sleep issues	3
Scar pain/adhesions	2
Fasciculation/motor dysfunction	1
Restless legs	1
Crohn’s disease	1
Total symptoms	60

**Fig. 4:**
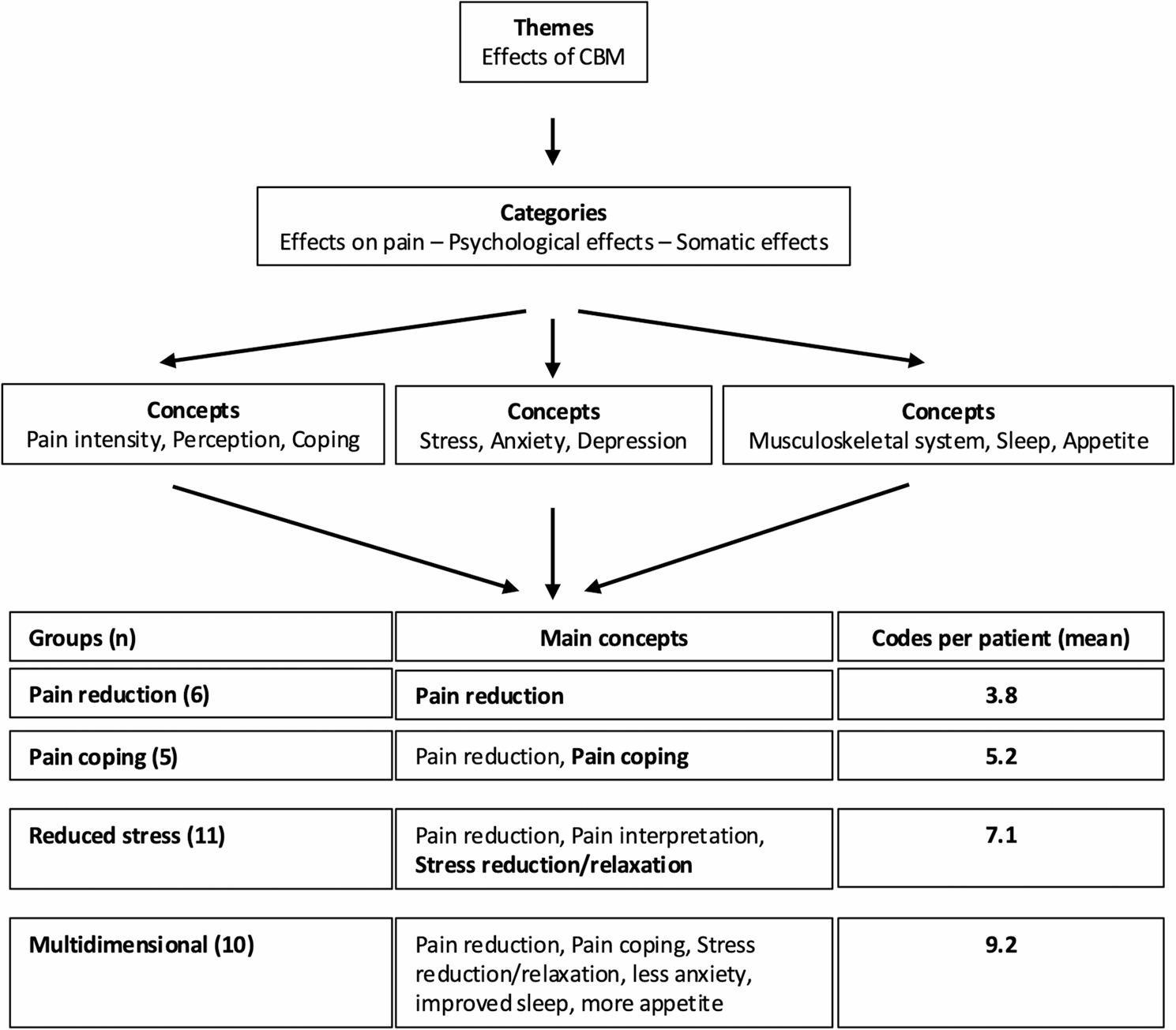
Grouping of 32 qualitative interviews in 4 different groups based on main concepts in each group using MAXDA (version 2022). Bold = Main concepts

After completing the coding process as described in the Methods section, our groups were identified based on the key areas that individual participants cited when asked what they had benefited from most as a result of CBM therapy (Fig. 4).

- Group 1 (6 patients) displayed mainly the concept of pain intensity and the main code of pain reduction.

“So, the logical thing is that I no longer have any pain. Not much has changed for me, as I still have other health problems. I no longer have any pain, which is worth a hell of a lot.” (ER018).

No changes in pain coping, distancing from pain, the perception of pain or the psychological effect were mentioned.

- Group 2 (5 patients) showed changes in all concepts related to the effect on pain, especially on pain coping and distancing from pain.

“So, the pain is there, but you can live with it, at least I personally can, if it stays like this, I can cope with the pain quite well.” (AB165).

Psychological effects were not reported by these patients.

- Group 3 (11 patients) showed changes in pain intensity and the perception of pain, as well as the interpretation of pain, but not in pain coping or distancing from pain. In addition, the patients described a psychological effect, especially in form of reduced stress. All patients in this group described experiencing relaxation and calming.

“And because of the cannabis drops you’re very relaxed, from the body. You know, the muscles have relaxed. You yourself are also sleepier. You yourself have mentally relaxed more.” (IR096).

- Group 4 (10 patients) displayed multidimensional aspects triggered by CBM-therapy adding to pain reduction, pain coping, and stress reduction also improvements in sleep, levels of anxiety, and appetite.

“So, I used to go to the bathroom three or four times a night […] because I just couldn’t lie down anymore. And now, out of seven nights, I really do sleep through five or six of them. […] You approach things very differently when you’re in a more positive frame of mind. Because when I used to get up in the morning, I would think: “Oh, not again today, and oh, and ah, everything hurts, and I don’t really feel like doing anything… and ahh.” […] Now you’re just… well, you’re not afraid of it anymore. And now it’s not a big deal for me anymore. I just do it without thinking about it beforehand. […] I can play and romp around with my children again. […] I can go to work. […] Before, I was at about a 7 or 8 on the pain scale. With cannabis, I’m at about a 2 or 3.” (EM 258).

Regarding the category of effect on pain, patients in group 2 and 4 described changes most often, with an average of 4.0 and 3.9 codes per patient, for example

“I would say that in the moment I’m inhaling it, during smoking, the pain is already, is already diminishing.” (OR085).

In contrast. in groups 1 and 3, the mean number of codes in this category was 1.6 and 2.35. This group-difference is reflected by the distribution in the concept of pain coping, where patients from groups 2 and 4 (on average 2.6 and 2.4 codes per patient) reported changes more often than patients in groups 1 and 3 (on average 0.3 and 0.72 codes per patient). In contrast, in the concept of pain intensity, in all groups mean codes per patient were between 1.0 and 1.3.

In the category of psychological effect, the average number of codes in groups 3 and 4 was similar, amounting to on average 2.17 and 2.2 codes per patient while in the groups 1 and 2 no psychological effect was observed.

Regarding the category of somatic effect, patients in group 4 reported the most changes with on average 3.1 codes per patient compared to the other groups. When observing the concepts of this category, it could be noted that patients reported more changes in most areas than patients of other groups, especially in improvements in muscle relaxation, sleep, and appetite.

The groups were further examined in relation to changes in the theme “QOL”. In this context, a relevant question was included in the semi-structured interview (supplementary data), and some patients spontaneously mentioned changes in their QOL during the interviews.

All patients experienced positive changes in QOL, stating for example

“It’s a gain in quality of life because of the diminished pain” (OU149).

or.

“When I sleep better, that’s a big win in quality of life.” (ET075).

When investigating the groups, it became apparent that patients in groups 2, 3 and 4 experienced changes in QOL more often than patients in group 1. In group 1, the average number of codes per patient was 3.3, which is much lower than in other groups (5 or more codes per patient).

In the last version of the interview guideline, which was used with participants 26–32, there were additional questions posed related to the mental relaxation ability of patients. Patients reported that before beginning the CBM therapy, they experienced problems with relaxing and switching off.

“(…) I never could just simply sit on the couch and relax. (…) I could always just power through.” (ET075).

or.

“I have always had problems with… quickly relaxing. But I think that’s kind of genetic, my mother was also like me.” (ER097).

After starting CBM therapy, participants reported a better ability to relax and calm down.

“And I can tell, that cannabis works in the right direction, namely letting go and relaxing…” (ET075).

These patients were assorted only in groups 3 and 4.

Looking at the results from the TSQM questionnaire (Table 3), it was found that approximately 94% of study participants achieved a score of ≥ 70 in terms of overall satisfaction, which is consistent with a high level of satisfaction with CBM. 59% even scored ≥ 90, which is consistent with an excellent rating of the therapy. This finding can be attributed to both high effectiveness (83% ≥70) and good tolerability (81% ≥80).

**Table 3:** Results of the German TSQM (Treatment Satisfaction Questionnaire for Medication)-questionnaire, version 1.4. In each category, a value between 0 and 100 is possible; the higher the number, the greater the patient satisfaction. List of the respective values per category and how many patients gave this rating (N). In the effectiveness category, only 29 patients were included in the evaluation, as an error was suspected in the responses of 3 patients (OL027, OR085, EM258). The patients gave very low scores for effectiveness, even though they appeared very convinced of the CBM therapy in the interview

Effectiveness										Total
Values	55.6	61.1	66.7	72.2	77.8	83.3	88.9	94.4	100	
N	2	1	2	1	8	4	3	3	5	29
Side Effects										
Values	50	56.25	68.75	81.25	87.5	93.75	100			
N	3	1	2	3	2	3	18			32
Convenience										
Values	66.7	77.8	83.3	88.9	94.4	100				
N	3	6	5	1	3	14				32
Global Satisfaction										
Values	57.1	66.6	71.4	78.6	85.7	92.9	100			
N	1	1	4	2	5	7	12			32

When connecting the results of the quantitative TSQM and the groups determined by the qualitative analysis (Table 4), it can be observed that patients in group 1 reported the lowest effectiveness of CBM, whereas group 4 reported the highest. Global satisfaction was lowest in group 2, which displayed the biggest changes in pain coping. The highest global satisfaction could be measured in group 4. Global satisfaction with CBM was also associated with a flexible intake schedule. A limited ability to drive due to the intake of CBM had a negative effect on global satisfaction.

**Table 4:** Effectiveness and global satisfaction in TSQM-questionnaire related to groups based on results of qualitative interviews (mixed methods). TSQM=Treatment Satisfaction Questionnaire for Medication (version 1.4), 0-100 (100 = maximal positive)

Groups based on results of qualitative interviews	Effectiveness (TSQM)	Overall satisfaction (TSQM)
Group 1 (pain reduction)	72.25	86.9
Group 2 (pain coping)	80	84.1
Group 3 (reduced stress)	81.8	91.6
Group 4 (multidimensional)	88.3	92.9

Most patients (29 out of 32) addressed during the interviews side effects spontaneously without being prompted.

Fifteen out of 32 patients did not report side effects in the TSQM, however, described cognitive impairment (*n* = 10), fatigue (*n* = 5), dry mouth (*n* = 4), dizziness (*n* = 2), inner tension (*n* = 2), and gastro-intestinal side-effects (*n* = 2) in the interviews.

A possible explanation for this discrepancy could be, that participants did not feel much impaired by the side effects and therefore did not report them in the quantitative TSQM. On the other side, 3 patients reported not feeling any side effects during the interviews but rendered high values in the TSQM.

It was also reported that after starting CBM therapy, other (pain) medications could be partially or completely discontinued.

“I had already tried several painkillers and now I still take metamizole from time to time, but I have been able to reduce my medication intake, including painkillers.” (AC219).

“But I think if this cannabis also relaxes me a little and so on, then it does have an effect, otherwise I would have to take more of the other pills again.” (OI054).

Some of our patients also reported that it was difficult to obtain access to medically prescribed CBM, sometimes involving a “struggle” that lasted several years.

“I think it should be easier for cannabis patients. Of course, it should be controlled and not available to everyone, depending on the illness. If you can really prove that it’s necessary. But […] making it so difficult for some people is […] bureaucracy […] unfortunately. […] What would be important for me, or for cannabis patients in general, is that all these costs […] that health insurance companies don’t want to cover […] that the process is made so difficult for patients in the first place, I think that really needs to be worked on.” (UE118).

Stigmatization was also discussed. Patients had negative experiences in the form of stigmatization by medical staff and their social environment. They experienced stigmatization when, for example, doctors refused to prescribe CBM because they did not want to treat “that type of clientele.” Some patients also conceal their therapy from those around them for fear of stigmatization.

“I was very disappointed when I realized how my environment reacted to my cannabis use. [laughs] Among my acquaintances, friends, and even at work. That you always get caught up in discussions and people don’t really understand. And in some cases, you get labeled as an addict or a junkie or something like that.” (EM258).

## Discussion

Qualitative interviews in this cohort showed that the effects of CBM on chronic pain are based on four different basic concepts: pain reduction (group 1) – pain coping (group 2) – stress reduction (group 3) – multidimensional aspects (group 4), with participants noticing and naming more effects in ascending order of the group-number. It was shown that as the number of different effects increased, so did QOL and overall satisfaction with treatment. Minor side effects were reported, mainly cognitive impairment, fatigue, and dry mouth, but these did not appear to affect the overall positive perception of CBM therapy.

This finding is consistent with the biopsychosocial model, which views chronic pain as a multidimensional, dynamic process in which biological, psychological, and social factors influence one another. According to this model, chronic pain can be considered a disease in its own right, and mental health conditions such as depression and anxiety can be not only a consequence but also a cause of pain. In contrast, the biomedical model assumes that while psychological and social factors influence pain, some form of tissue damage must always be present. The biomedical model is now considered outdated. The biopsychosocial model is widely recognized as an explanation for the development of chronic pain, as evidenced by the definition of chronic pain provided by the International Association for the Study of Pain (IASP) (Treede et al. 2019, Barke et al. 2022, Nijs et al. 2021).

A qualitative study conducted by an Israeli group on 19 patients with chronic pain showed that patients benefit from CBM primarily by regaining control over their everyday lives and being able to take on an active role again. This enabled them to be themselves again, i.e., to regain a sense of normality (Lavie-Ajayi and Shvartzman 2019).

These results largely correspond with our findings. However, by exploiting survey saturation and using a mixed-methods approach, we were able to show that this holistic benefit can be achieved more effectively if CBM has a broad effect on both somatic and psychological levels, which is not the case for every patient. The reasons for this may lie primarily in the individual spread of the ECS in the central nervous system (CNS) or in the extent of ECS activity in the dysfunctional connectome, which can be understood as a central correlate of chronic pain (Schaffer et al. 2023, Vachon-Presseau et al. 2016) and which also overlaps to a large extent with the stress system (Schaffer et al. 2023, Timmers et al. 2019) and can also be directly attenuated by THC (Weizman et al. 2018).

Our observation that patients who described the most pronounced multidimensional effects benefited most from CBM therapy could be understood to mean that there are positive correlations between the immediately experienced somatic and psychological effects and the pain affect. One could also speak of an embodiment effect due to the effects of CBM, i.e., positive bodily phenomena are experienced and reflected on a psychological level, which can lead to a realignment of the entire person. The fact that the overall effect of the treatment is more significant than the mere pain-reducing effect of CBM was also demonstrated in a recently published RCT, in which more than 800 patients with chronic low back pain (cLBP) received either a full cannabis extract or a placebo. The NNTB for pain reduction was 6.8, and the NNTB for the clinical global impression (CGI) was 4.6 (Karst et al. 2025). As a result of these findings, the success of CBM treatment should be evaluated based on various factors, both in clinical practice and in clinical trials.

In their interview cohorts of 11 to 13 patients with chronic pain, authors Luque et al. (Luque et al. 2021) und AminiLari et al. (AminiLari et al. 2022) also found that CBM can lead to decreased pain, improved mood, improved QOL, improved sleep, and improved appetite/energy. Similar to our work, these publications showed that for a small proportion of participants, other medications could be reduced or eliminated, and that costs, access to CBM, and stigma associated with CBM were significant barriers. An online survey of more than 1,500 respondents who used CBM primarily for chronic pain (70.4%) and PTSD (25.5%) provided at least indirect evidence that CBM has a broad impact across various areas (Piper et al. 2017). The majority of respondents who regularly took medication for various conditions reported that they were able to reduce their doses of opioids as well as medications for anxiety, migraines, and sleep disorders (Piper et al. 2017). A systematic review of qualitative studies on the experiences of patients using CBM for pain management identified four overarching key themes across the eight studies included in the analysis (Ng et al. 2022): potential benefits; patients’ experimentation with cannabis and their knowledge of it; problems associated with cannabis use; and physicians’ lack of experience with cannabis in pain management. Regarding the benefits, patients primarily reported pain relief and improvements in sleep, fatigue, drowsiness, and nausea, among many other aspects. The study also showed that physicians rarely provided their patients with reliable scientific evidence, which led patients to experiment with cannabis (Ng et al. 2022). Regarding problems associated with cannabis use, psychophysical side effects such as increased respiratory problems and anxiety, as well as the stigma associated with use and high retail prices, were reported (Ng et al. 2022). Although patients in our cohort also expressed concerns about barriers to accessing information about CBM and the therapy itself, as well as stigma and costs, these issues were not the primary focus. All of our patients received CBM as a prescription medication under medical supervision, with costs covered by health insurance. Furthermore, most of our patients used oral CBM preparations or, in the case of inflorescences, a vaporizer. This framework appears to foster a positive and reassuring attitude toward CBM as a beneficial component of the treatment of their chronic pain symptoms.

### Strengths and limitations

We applied a rigorous qualitative methodology to investigate the attitudes of people with chronic pain toward CBM and recruited a comparatively large number of patients for qualitative interviews (32 participants) (Moser et al. 2018, Grossoehme 2014) to achieve sufficient thematic saturation. In addition, we combined the TSQM questionnaire with the qualitative interviews to integrate the quantitative and qualitative findings in a way that complemented one another.

The monocentric characteristic of our study can be viewed as a limitation, but in qualitative research, the selection of participants is primarily concerned with the specificity of the sample, as was the case in the Pain Clinic at Hannover Medical School. This was the only way to ensure that the patients took CBM as prescribed by their physician.

The qualitative methodology itself could be viewed as a limitation, since no specific hypothesis was tested and the analysis was primarily descriptive. However, this is consistent with qualitative research and is a core principle of the grounded theory approach used here, in which the groups were formed inductively. The goal of this methodology is to gain insights into a topic without having a specific hypothesis in mind. Our intention was not to measure efficacy (e.g., by measuring pain intensity), but rather to determine, from the perspective of these patients, why CBMs are effective. Therefore, we specifically surveyed only those patients who appeared to benefit from CBM therapy and did not include a comparison group. In our cohort, the ratio of participants who received synthetic CBM to those who received non-synthetic CBM was highly uneven. Given the small overall sample size and the qualitative approach, a classical statistical analysis was not planned and was not conducted. Such tests would be of very limited validity and would rather carry the risk of overinterpretation. Also the TSQM component was used to descriptively characterize the qualitatively formed groups as part of an exploratory mixed-methods comparison; it is therefore more a matter of a complementary integration of quantitative and qualitative findings than a formal hypothesis testing. Particularly in qualitatively driven designs, the quantitative component is frequently used descriptively to illustrate patterns, not to test for differences using inferential statistics (Fetters et al. 2013). We hope that the results of our study will help identify appropriate parameters that should be taken into account when measuring the efficacy of CBM in future randomized controlled trials.

Other limitations include volunteer, selection, recall, and social desirability bias on the part of patients, and researcher and confirmation bias on the part of investigators.

However, all the points mentioned above presented a low risk: of the 43 patients who were eligible and approached, 42 agreed to participate. The selection of patients (prescribed CBM for at least 6 months) was based on the study question. The high number of respondents meant that the risk of recall bias was kept to a minimum. The interviews were conducted by FraF, who was not involved in the treatment of the patients. The risk of researcher bias and confirmation bias was kept low through the exclusive use of open-ended questions, the use of MAXQDA^®^, separate evaluations, extensive discussions within the study team, and mixed methods.

Another limitation concerns patient selection, as only patients who had undergone treatment for at least six months were included. This approach resulted in the inclusion of patients who tolerated CBM well, while excluding those who experienced intolerable side effects or did not perceive any benefit and discontinued CBM prematurely. The overall study design was accordingly tailored to include only patients with chronic pain who demonstrated a benefit from CBM.

## Conclusion

If the effects of CBM therapy are not only evident in terms of pain relief but also multidimensional, particularly in pain coping (including pain interpretation), stress reduction (including muscle relaxation), anxiety reduction, improved sleep, and increased appetite, a stronger effect in terms of pain relief and QOL can be expected.

This may be associated with the targeting of CB1 receptors in the cortico-mesolimbic system and a close interrelationship between somatic and psychological experience triggered by CBM therapy.

As this is primarily a qualitative study, these results should not be interpreted as prospective data-based evidence, nor should they be confused with such evidence. Rather, the study provides valuable information about the subjective changes that can be achieved with CBM in patients with chronic pain. An association was found between the multidimensionality of these changes and the extent of the treatment outcome.

This information can be used for future prospective clinical trials, e.g., in the selection of a holistic composite outcome, such as the holistic minimal clinically important differences (MCID) capturing treatment responses across 5 different outcome domains proposed by Taylor et al. (Taylor et al. 2024).

## Supplementary information

Supplementary Material 1.

## Data Availability

The datasets generated and/or analyzed during the current study are not publicly available due to data protection and privacy policies but are available from the corresponding author on reasonable request.

## Abbreviations

CB: Cannabinoid
CBM: Cannabis based medicines
CGI: Clinical global impression
cLBP: Chronic low back pain
CNCP: Chronic non-cancer pain
CNS: Central nervous system
ECS: Endocannabinoid system
IASP: International Association for the Study of Pain
MCID: Minimal clinically important differences
NNTB: Number needed to treat for benefit
PTSD: Post-traumatic stress disorder
QOL: Quality of life
SRMA: Randomized controlled trial
SRMA: Systematic review and meta-analysis
THC: Tetrahydrocannabinol
TSQM: Treatment Satisfaction Questionnaire with Medication
