# Supplementary material for: The greater the pleiotropic effects, the greater the benefits - cannabis as a “biopsychosocial” drug: a mixed-methods study on chronic non-cancer pain
Source: J Cannabis Res. 2026 Apr 21;8:54. doi: 10.1186/s42238-026-00440-w (PMC13097855; doi:10.1186/s42238-026-00440-w)
Supplement: Supplementary file 1 — Supplementary Material 1. [file 42238_2026_440_MOESM1_ESM.docx]

Supplemental data

**Interview guideline, first version (patients 1-25)**

1. Who had the initiative to start therapy with a cannabis-based medication?

a) If the initiative started from you

I. What was the reasoning for your initiative?

If necessary, present examples: media coverage, self-experience with non-medical cannabis

II. How difficult did the way to therapy present?

2. Why was a cannabis-based medication prescribed to you?

3. What were your expectations from the cannabis-based medications when it was prescribed to you?

4. How were your expectations met?

5. How were your expectations not fulfilled?

6. What changed for you since the beginning of the cannabis-based medication?

7. How would you describe the effect of the cannabis-based medication?

If necessary, ask further: stress, tension, psychological load, trouble falling or staying asleep, appetite

1. If you have previous experience with non-medical cannabis: what is the difference?
2. If you have experience with different cannabis-based medication: how would you describe the different effects on your symptoms?

8. What influence does it have on your day-to-day life?

9. How and in which way did your quality-of-life change since beginning treatment with the cannabis-based medication?

10. You are a patient in the pain clinic: How does the cannabis-based medication influence your pain?

11. Do you have anything else you want to add to this topic?

**Interview guideline, second version (patients 26-32)**

1. Who had the initiative to start therapy with a cannabis-based medication?

a) If the initiative started from you

I. What was the reasoning for your initiative?

If necessary, present examples: media coverage, self-experience with non-medical cannabis

II. How difficult did the way to therapy present and what was your experience with the different actors of the health system?

e.g. doctors, pharmacists, health insurance, police

2. Why was a cannabis-based medication prescribed to you?

3. What were your expectations from the cannabis-based medications when it was prescribed to you?

4. How were your expectations met or not fulfilled?

5. What changed for you since the beginning of the cannabis-based medication?

6. How would you describe the effect of the cannabis-based medication?

If necessary, ask further: stress, tension, psychological load, trouble falling or staying asleep, appetite

1. If you have previous experience with non-medical cannabis: what is the difference?
2. If you have experience with different cannabis-based medication: how would you describe the different effects on your symptoms?

7. What influence does it have on your day-to-day life?

8. How and in which way did your quality-of-life change since beginning treatment with the cannabis-based medication?

9. You are a patient in the pain clinic: How does the cannabis-based medication influence your pain?

10. How difficult is it for you, generally speaking, to relax or to switch off, even independently from pain?

Present examples: from work, set tasks and goals

11. Within your pain disease, did you have any support from psychological or behavioral therapeutic methods?

Examples: Talking therapy, progressive muscle relaxation according to Jakobson, meditations, autogenic training, yoga

1. how did this help you?
2. Which parallels do you see between these programs and the effects of the cannabis-based medication?

12. Do you have anything else you want to add to this topic?

Table S1. Sociodemographic baseline characteristics of the participating patients, including background medications.

Table S2. Type of coding themes and example phrases.

| Overarching Phenomenon | | Example Phrases |
| --- | --- | --- |
| Pain Perception During CBM Therapy | |  |
|  | Themes |  |
| Underlying  Conditions | Prescription | „Yes, I had tried all the medications, and basically that meant I had exhausted all treatment options.“ (RJ116)  „I just felt sick; I wasn't human anymore.“(ER097)  „And I thought that might be a little smoother.“ (EL055)  „Yeah, and then I did a little research… about cramps and nerve pain, and that’s how I came across cannabis on the internet.” (OR115) |
|  | Initial State | „I had really severe pain throughout my entire leg. If you use a pain scale from 1 to 10, I said I was at 14 or 17. I couldn’t even put a number on it anymore.” (RJ116)  „Because at some points it was really that bad. I was desperate, to be honest.” (EL055)  „Because when I don’t sleep well, the pain gets worse.” (EL055) |
| Context | Healthcare System | „Many doctors also said, ‘Yes, but you’ll become dependent on it. That’s … bad and … terrible. I wouldn’t recommend it.’” (AV189)  „After two years, I finally had what you might call permanent permission, and my health insurance was covering the costs as well.” (AC219)  „At some point, I had a prescription and ended up sitting in a parking lot somewhere, crying, because I couldn’t find a pharmacy. That was the next ordeal.” (RJ116) |
|  | Factors that have influenced patients' attitudes toward CBM | „I was really disappointed when I realized how the people around me react to the fact that I use cannabis. Like among people I know, friends, and even at work. That you keep getting squeezed into these discussions and people just don’t really get it. And sometimes you get labeled as an addict or a junkie or something like that.” (EM258) |
|  | Coping Strategies | „I’ve been doing yoga since I was a teenager. […] It’s always helped me, it helps me now, and I wouldn’t want to be without it—or the physical therapy I’m receiving.” (AR115)  „Well, I’d say: Yeah, sure, there are parallels… definitely. Because both of them [CBM and relaxation methods] have a bit of a relaxing effect.” (AV189) |
| Intervening Conditions | User experiences of CBM Therapy | „So there are no side effects, except maybe a dry mouth once in a while.” (TI286)  „At first, it was just the usual stuff with short-term memory impairment. […] But that eventually went away.” (ER018)  „And for the past six months, I haven’t taken anything at all. […] The pain has gradually subsided, so it’s been good for me in terms of relief – you could say it’s helped me manage it. And the few times I still get headaches now, they’re bearable.” (OL027)  „Well, it really works well. It’s fast, too – like right away… you notice it immediately when you start, so there’s definitely a quick improvement.” (ES116)  „My pain isn’t – well, the pain is still there, but I feel it very differently. I feel more relaxed.” (AR115)  „I don’t need a walking stick anymore either.” (AB165) |
| Effect | Effects of CBM Therapy on Different Domains | „So the pain is there, but you can – with this pain – well, personally, if it stays this way, I can cope with the pain just fine.” (AB165)  „Because I just can’t anymore – I don’t feel so limited anymore. This allows me to participate in life.” (UE118)  „And then – this might sound strange – but then I can just let myself drift off to sleep.” (RJ116) |
| Consequence | Impact of CBM on QOL | „I...am basically back to being more or less the person I was before the pain.” (CC147)  „It was such a relief – you can’t even imagine.” (AL235) |
